# Active particles crossing sharp viscosity gradients

**DOI:** 10.1038/s41598-023-27423-8

**Published:** 2023-01-11

**Authors:** Jiahao Gong, Vaseem A. Shaik, Gwynn J. Elfring

**Affiliations:** 1 Department of Mathematics, University of British Columbia, 1984 Mathematics Road, Vancouver, BC V6T 1Z2 Canada; 2 Department of Mechanical Engineering, Institute of Applied Mathematics, University of British Columbia, Vancouver, BC V6T 1Z4 Canada

**Keywords:** Biological physics, Fluid dynamics

## Abstract

Active particles (living or synthetic) often move through inhomogeneous environments, such as gradients in light, heat or nutrient concentration, that can lead to directed motion (or *taxis*). Recent research has explored inhomogeneity in the rheological properties of a suspending fluid, in particular viscosity, as a mechanical (rather than biological) mechanism for taxis. Theoretical and experimental studies have shown that gradients in viscosity can lead to reorientation due to asymmetric viscous forces. In particular, recent experiments with *Chlamydomonas Reinhardtii* algae swimming across sharp viscosity gradients have observed that the microorganisms are redirected and scattered due to the viscosity change. Here we develop a simple theoretical model to explain these experiments. We model the swimmers as spherical squirmers and focus on small, but sharp, viscosity changes. We derive a law, analogous to Snell’s law of refraction, that governs the orientation of active particles in the presence of a viscosity interface. Theoretical predictions show good agreement with experiments and provide a mechanistic understanding of the observed reorientation process.

## Introduction

Active particles are living or non-living entities that convert stored energy to directed motion and a suspension of these particles is termed active matter^1^. Examples of active particles range from nanorobots and microorganisms to birds, fish and even humans^2,3^. Our focus here is on micron-sized active particles that move through a viscous fluid such that inertia is negligible. Active particles at this scale exhibit rich phenomena like boundary accumulation^4,5^, upstream swimming^6–10^, collective motion^11^, active turbulence^12^ and motility-induced phase separation^13^.

Active particles often move through inhomogeneous environments with spatial gradients in light^14^, heat, nutrient concentration or other chemical stimuli^15^. These spatial gradients in their environment can affect the dynamics of active particles and lead to directed motion (or *taxis*). Taxis can be an active response, as particles sense the local gradients and actively change their motion. Examples include *E. coli* which prolongs runs when swimming up nutrient gradients to pursue nutrient-rich regions^16,17^. On the other hand, taxis can be a passive response, caused solely by physical interaction with the environment that modifies particle dynamics. Examples of this sort include the chemotactic behavior of Janus particles^18,19^ and active droplets^20,21^. Inhomogeneous environments can also be leveraged to sort or organize active particles. For example, the photophobic response of *E. coli* can be used to ‘paint’ with the bacterium by subjecting a bacterial suspension to light gradients^22,23^. Recent research has explored inhomogeneities in the rheological properties of fluids (such as viscosity^24,25^, or viscoelasticity^26,27^) as a mechanical (as opposed to chemical or biological) mechanism of spatial control and taxis.

Viscosity gradients are prevalent in mucus layers^28^, oceans, lakes, and ponds^29^, mostly caused by similar changes in temperature, salinity or nutrient concentrations. The average viscosity of mucus ranges from 2 cP-1000 cP depending on the organism^30,31^, while that of oceanic waters is 1.070 cP^32^ and the corresponding gradient in the vicinity of phytoplankton is $0.015\ \text {cP}/\upmu \text {m}$^33^. In such gradients, particles tend to perform taxis by moving up or down the gradients (defined as positive and negative viscotaxis respectively). For instance, organisms like *Leptospira* and *Spiroplasma* have been observed to perform positive viscotaxis^34–37^ while *E. coli* has been observed to perform negative viscotaxis^38^. Recent experiments with *Chlamydomonas Reinhardtii* show contrasting behavior in weak vs strong gradients^24^. In weak gradients, the algae accumulate in high viscosity regions due to their low speed but in strong gradients, they reorient to move towards low viscosities (negative viscotaxis).

**Figure 1:**
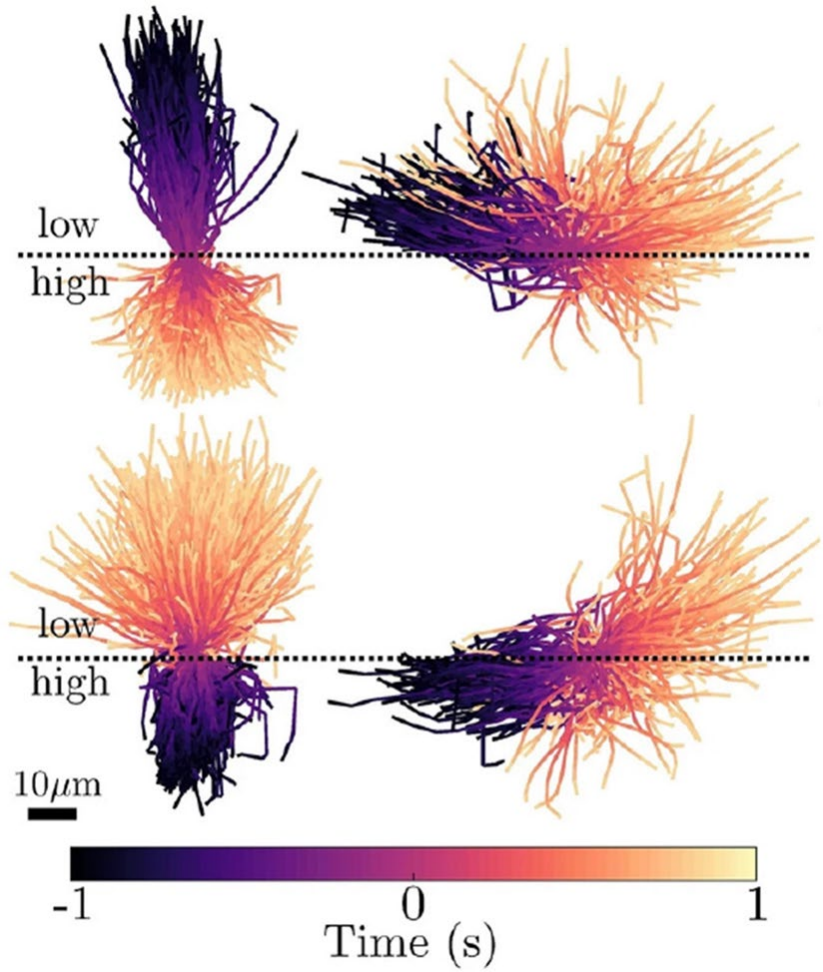
Trajectories of *Chlamydomonas Reinhardtii* going from low to high (top) and high to low (bottom) viscosities from recent experimental work^25^. The trajectories on the left are relatively steep (closely aligned to the interface-normal) while those on the right are relatively shallow and display scattering at the interface when going from low to high viscosities. Image is from the paper by Coppola and Kanstler^25^ which is licensed under https://creativecommons.org/licenses/by/4.0/CC BY 4.0.

A simple fluid mechanical mechanism for viscotaxis was developed by modeling active particles as connected spheres driven by a fixed propulsive force in weak viscosity gradients^39^. Such particles exhibit taxis due to a systematic mismatch of viscous drag acting on different spheres leading to a torque that generally reorients particles to move up viscosity gradients^39^. Subsequent work modeled active particles as spherical squirmers, where the particle activity that generates thrust is included but abstracted to a representative surface slip, which is a particularly apt representation of active particles that do not undergo large changes of geometry such as diffusiophoretic Janus particles and ciliated organisms like *Paramecium* or *Opalina*. In this case the interaction of the spatially varying viscosity with the active slip boundary conditions on a squirmer generically resulted in negative viscotaxis^40,41^. A different swimmer, Taylor’s swimming sheet speeds up while moving along or against the gradients^42^. These theoretical models all rely on weak, diffuse gradients in the fluid viscosity. However recent experiments have shown very interesting particle dynamics in sharp viscosity gradients both for synthetic^43^ and biological active particles^25^. In particular, we are interested here in experiments which probed the motion of *Chlamydomonas Reinhardtii* swimming across a sharp jump (or interface) in viscosity between miscible fluids^25^. Among other results, it was found that the algae would be quickly reoriented by the interface in viscosity and if the organism approached the interface at a sufficiently shallow angle could be reflected by the interface if going from low to high viscosity (see Fig. 1). Here, we develop a simple fluid dynamical model to unravel the physics underlying these experiments. We model the swimmers as spherical squirmers and focus on small, but sharp, viscosity changes. We show that the reorientation process is always in the direction of lower viscosity and derive a law, analogous to Snell’s law of refraction, that governs the orientation of active particles in the presence of a viscosity interface. In analogy to ray optics, the refraction of the trajectory is always towards the medium of lower resistance. As we will show below, our theory (for pullers) matches well with experimental observations of *Chlamydomonas Reinhardtii* algae swimming across sharp viscosity gradients^25^. Our results are also quite similar to recent theoretical work modeling gliders moving across a substrate features a jump in frictional properties. In particular, the functional form of the reorientation law we find is identical to that found for gliders^44^. However that work, and other studies where the propulsive force is similarly fixed^39^, shows reorientation towards higher viscosities as one might expect due to the modulation of drag alone.

We organize the paper as follows. In the following section, we provide the essential details of our model and the resulting particle dynamics. We then interpret the implications of our model and compare them with experimental observations in “Results” section. We then provide some concluding remarks and finally the technical details concerning the mathematical methods used are left to “Methods” section.

## A model for active particles crossing sharp viscosity gradients

We consider an active particle immersed in an otherwise quiescent fluid, moving near and across a region where the fluid has a relative sharp change of viscosity. This change in viscosity can be due to a corresponding variation in fluid temperature, salinity, or a nutrient dissolved in the fluid. Regardless of the origin, one expects sharp viscosity gradients to vanish due to diffusion over long times, but during the short time scales over which the particle crosses the interface, relatively sharp gradients can be stable^25^. For instance, *Chlamydomonas Reinhardtii* algae, with a characteristic size of $\approx 10 \,\upmu$m, traveling at a body length per second take $O(10\,{\textrm{s}})$ to approach and cross the interface while the salinity gradients take $O(10^3\,{\textrm{s}})$ to vanish^25^. We assume that the fluid viscosity is prescribed and steady, and not significantly disturbed by the presence and activity of the moving particle as in previous work^39–41^; however, this is an uncontrolled approximation because we assume that the sharp gradient persists, nevertheless we will show that this reasonably captures the experimental observations^25^. We note that the viscosity gradients considered here are distinct from the long-lasting viscosity differences that can exist at the interface between immiscible fluids with non-negligible surface tension that can dramatically affect (and even prevent) the particle crossing^45–48^.

**Figure 2:**
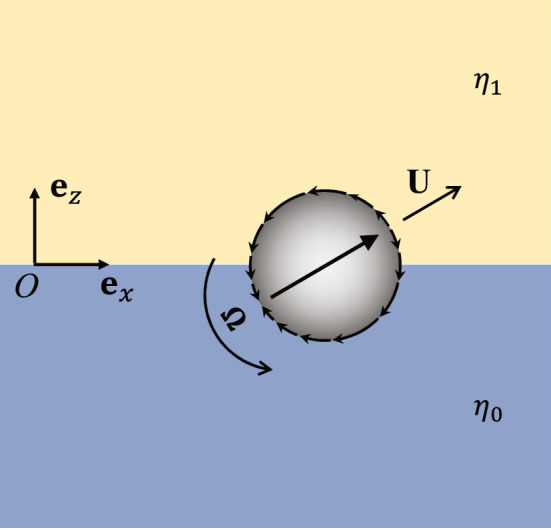
Schematic showing an active particle passing through sharp viscosity gradient and the associated coordinate system. The interface separates fluid of different viscosities $\eta _0$ and $\eta _1$. The particle radius is *a* and its translational and rotational velocities are ${\textbf{U}}$, $\varvec{\Omega }$, respectively.

For simplicity we assume changes in only one direction and choose a coordinate with the $z-$axis oriented in the direction of change, such that $\eta = \eta (z)$. The viscosity changes from one uniform viscosity $\eta (z\rightarrow -\infty ) = \eta _0$ to another $\eta (z\rightarrow \infty )=\eta _1$, and define a relative change in viscosity $\varepsilon = \left( \eta _1 - \eta _0 \right) /\eta _0$. We will first assume the viscosity jumps (discontinuously) from $\eta _0$ to $\eta _1$ at $z=0$, as shown in Fig. 2. In this case the viscosity field may be written

1 $$\begin{aligned} \eta (z) = \eta _0\left[ 1 + \varepsilon H(z)\right] , \end{aligned}$$

where *H*(*z*) is the Heaviside function. This representation is of course an idealization (as finite diffusivity would instantly smooth any discontinuity); however, we will later show that relaxing this assumption to smooth changes in viscosity leaves our results unchanged. To make mathematical progress we focus on small changes in viscosity such that $\left| \varepsilon \right| \ll 1$, but we believe the main physical picture holds for any $\varepsilon$.

The fluid flow generated by the active particle satisfies the incompressible Stokes equations

2 $$\begin{aligned} \varvec{\nabla } \cdot \varvec{\sigma } = {\textbf{0}}, \quad \varvec{\nabla } \cdot {\textbf{u}} = 0, \end{aligned}$$

where ${\textbf{u}}$ is the velocity field and stress in a Newtonian fluid, $\varvec{\sigma } = - p {\textbf{I}} + \eta \dot{\varvec{\gamma }}$, where *p* is the pressure, $\dot{\varvec{\gamma }} = \nabla {\textbf{u}} + (\nabla {\textbf{u}})^T$ is the fluid strain-rate tensor.

The active particle swims with a translational velocity ${\textbf{U}}$ and an angular velocity $\varvec{\Omega }$ due to its activity. Thus, the velocity of the fluid on the surface of the particle $S_p$, can be decomposed as

3 $$\begin{aligned} {\textbf{u}}({\textbf{x}}\in S_p) = {\textbf{U}} + \varvec{\Omega } \times {\textbf{r}} + {\textbf{u}}^s, \end{aligned}$$

where ${\textbf{u}}^s$ is the boundary velocity of the activity alone, ${\textbf{r}} = {\textbf{x}}-{\textbf{x}}_c$, and ${\textbf{x}}_c = \left( x_c, y_c, z_c\right)$ denotes the particle (center) position and $\dot{{\textbf{x}}}_c={\textbf{U}}$. Far from the particle the fluid remains quiescent, hence

4 $$\begin{aligned} {{\textbf{u}}} \rightarrow {\textbf{0}} \qquad \text {as} \,|{{\textbf{r}}}| \rightarrow \infty . \end{aligned}$$

Here we prescribe the activity of the particle ${\textbf{u}}^s$, but the translational and rotational velocity are fixed by the dynamic constraints on the particle. The particle is inertialess and neutrally buoyant and with no external forcing acting on it therefore the hydrodynamic force and torque on the particle must vanish

5 $$\begin{aligned}{}&{\textbf{F}} = \int _{S_p} {\textbf{n}}_p \cdot {\varvec{\sigma }} \, dS = {\textbf{0}}, \end{aligned}$$

6 $$\begin{aligned}{}&{\textbf{L}} = \int _{S_p} {\textbf{r}} \times ({\textbf{n}}_p \cdot {\varvec{\sigma }}) \, dS = {\textbf{0}}, \end{aligned}$$

where ${\textbf{n}}_p$ is a unit normal to the particle surface.

Specifically, we model the active particle as a spherical squirmer of radius *a*. In the squirmer model, the details of the surface activity of the swimmer are coarse-grained into a prescribed tangential slip velocity on the surface of a spherical particle^49–51^. This model is particularly well-suited for ciliated microorganisms like *Paramecium* and *Opalina* that propel by synchronously beating numerous very small cilia on their surface, or diffusiophoretic Janus particles, which propel due to the motion of a thin layer of fluid on their surface as a result of chemical gradients^15^. The slip velocity is generally decomposed into Legendre polynomials (called squirming modes) in the form

7 $$\begin{aligned} {\textbf{u}}^s = \sum _{n=1}^{\infty } \frac{2 B_n}{n(n+1)} P_n'\left( \textbf{p} \cdot {\textbf{n}}_p\right) \textbf{p} \cdot ({\textbf{I}} - {\textbf{n}}_p {\textbf{n}}_p ), \end{aligned}$$

where $\textbf{p}$ is the particle orientation, $P_n$ is the Legendre polynomial of degree *n* and $B_n$ represents the coefficients of the squirming modes. In homogeneous Newtonian fluids, the $B_1$ mode alone determines the swimming velocity (we assume here $B_1>0$), whereas $B_2$ mode is the slowest decaying contribution to the far-field flow, furthermore the second mode determines whether propulsion is primarily from the front or the back of the swimmer. Organisms, such as *E. coli*, which produce propulsion from their rear end are called pushers, have $B_2 < 0$ whereas those that pull the fluid in front of them using their flagella are called pullers, such as *Chlamydomonas Reinhardtii*, and have $B_2 > 0$. Swimmers, with propulsion that is not distinctly fore or aft, such as *Volvox carteri* with flagella uniformly distributed on its surface, are called neutral and are well described by setting $B_2 = 0$. Here we look at only the effects of the first two modes, and while one is generally only well justified in neglecting higher-order modes in the far-field, we find dynamics here to be well captured by just the $B_1$ mode.

The fluid flow field and particle velocity can be determined simultaneously by solving the Stokes equations for a force and torque-free particle. Here, we take a perturbative approach; when $\varepsilon \rightarrow 0$ the viscosity is uniform and the solution to a single squirmer is well known, we then obtain the leading order correction in terms of the viscosity jump $\varepsilon$, by means of the reciprocal theorem (see “Reciprocal theorem” subsection in “Methods” for more technical details).

At the leading order in $\varepsilon$, the particle moves through a homogeneous Newtonian fluid of viscosity $\eta _0$ and its velocity is well known^49,50^

8 $$\begin{aligned} {\textbf{U}}_0 = \frac{2}{3}B_1 {\textbf{p}}, \,\, \varvec{\Omega }_0 = {\textbf{0}}. \end{aligned}$$

The viscosity variations relative to $\eta _0$ are captured at the next order and the particle swims due to these variations at velocity

9 $$\begin{aligned} {\textbf{U}}_1&= B_1 \left[ A(z_c) {\textbf{n}} + B(z_c) {\textbf{p}} \right] + B_2 \left[ C(z_c) {\textbf{n}} + D (z_c) (\textbf{pp} \cdot {\textbf{n}}) + E (z_c) ({\textbf{n}} \cdot {\textbf{p}})^2 {\textbf{n}} \right] , \end{aligned}$$

10 $$\begin{aligned} \varvec{\Omega }_1&= \left[ B_1 f(z_c) + B_2 g(z_c) \left( {\textbf{n}} \cdot {\textbf{p}} \right) \right] ({\textbf{n}} \times {\textbf{p}}), \end{aligned}$$

where we have assumed a regular expansion in $\varepsilon$, ${\textbf{U}}(\varepsilon ) = {\textbf{U}}_0+\varepsilon {\textbf{U}}_1+O(\varepsilon ^2)$, and $\varvec{\Omega }(\varepsilon ) = \varvec{\Omega }_0+\varepsilon \varvec{\Omega }_1+O(\varepsilon ^2)$. Here ${\textbf{n}} = {\textbf{e}}_z$ is the interface normal pointing from fluid of viscosity $\eta _0$ to that of viscosity $\eta _1$ while the functions $A \left( z_c \right)$, $B \left( z_c \right)$, $C \left( z_c \right)$, $D \left( z_c \right)$, $f\left( z_c \right)$, and $g\left( z_c \right)$ depend on the particle’s separation from the interface and are given in “Methods” section. The piecewise behavior of these functions and of the velocities $\mathbf{{U}}_1$, $\varvec{\Omega }_1$ is due to the fact that the particle is in contact with the viscosity interface when $\left| z_c \right| \le a$ and otherwise not. It is important to note that for $\left| z_c \right| > a$ the particle is still affected by the presence of the viscosity change due to hydrodynamic interactions mediated by the fluid at a distance.

We show later that the reorientation caused by $\varvec{\Omega }_1$ is always towards the low viscosity fluid. The physical reason for this is similar to that observed in weak gradients^40^. Both the rigid-body drag felt by the particle and the propulsive thrust generated by surface activity are altered by the presence of viscosity changes in the fluid (both due to contact and hydrodynamic interactions). Changes in the thrust tend to cause rotation towards regions of lower viscosity, while the opposite is true for changes to the drag, and in all instances the former dominates the dynamics (for spherical squirmers). The speed changes represented by $\mathbf{{U}}_1$ here are somewhat more complex than those observed in weak gradients, with even the neutral swimmer speeding up or slowing down depending on its position and orientation relative to the interface. However, these speed changes do not affect leading order trajectory of the particle.

In order to quantify particle reorientation, we simply integrate particle velocities. Noting that $\dot{{\textbf{x}}}_c={\textbf{U}}$ and $\dot{{\textbf{p}}}=\varvec{\Omega } \times {\textbf{p}}$, we substitute the leading order results for the particle velocities and project onto the interface normal direction ${\textbf{n}}={\textbf{e}}_z$ to obtain

11 $$\begin{aligned} \frac{d z_c}{dt}&= \frac{2}{3}B_1 \cos \theta + O\left( \varepsilon \right) , \end{aligned}$$

12 $$\begin{aligned} \frac{d\theta }{dt}&= \varepsilon \left( B_1 f\left( z_c\right) + B_2 g\left( z_c\right) \cos \theta \right) \sin \theta + O\left( \varepsilon ^2\right) , \end{aligned}$$

where the angle between the particle direction and the interface is defined by ${\textbf{p}}\cdot {\textbf{n}}=\cos \theta$. Combining these equations gives

13 $$\begin{aligned} \frac{d\theta }{d \,z_c} = \frac{3}{2}\varepsilon \bigl ( f(z_c) + \beta g(z_c) \cos \theta \bigr ) \tan \theta + O\left( \varepsilon ^2 \right) , \end{aligned}$$

where $\beta = B_2/B_1$. This differential equation entirely captures the leading order effect on particle orientation $\theta$ of a viscosity jump at a distance $z_c$ from the particle center. We note that any effect of viscosity on the translational velocity of the particle would enter at $O\left( \varepsilon ^2\right)$ in (13) and so is negligible compared to the leading order terms for $\varepsilon \ll 1$. As we will show, Eq. (13) is straightforward to integrate analytically for neutral squirmers, $\beta =0$, and easily integrated numerically for pushers ($\beta < 0$) and pullers ($\beta > 0$), and the rest of this paper are results and discussion that arise out of it.

## Results

We begin first with analytical results for neutral squirmers, $\beta =0$, before proceeding to present numerical results for pushers ($\beta < 0$) and pullers ($\beta > 0$) and a comparison to recent experiments for pullers.

**Figure 3:**
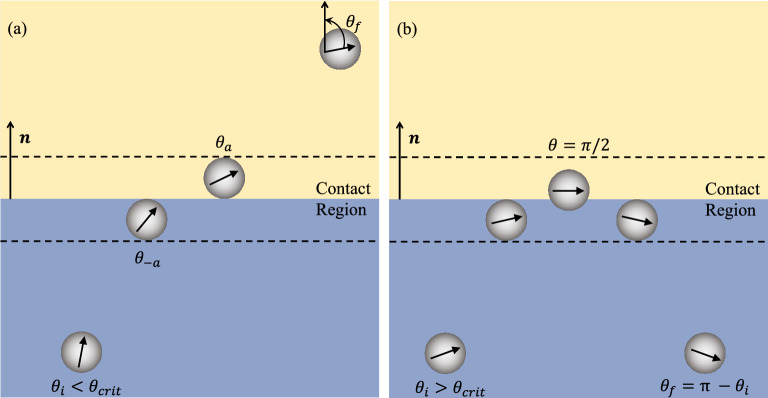
Schematic showing the reorientation of an active particle as it ($\textbf{a}$) crosses a viscosity interface or ($\textbf{b}$) gets reflected by the interface. This reorientation depends largely on the viscosity difference $\eta _1-\eta _0$ and the particle is reflected only when going from low to high viscosity if its initial orientation is sufficiently shallow, $\theta _i > \theta _{crit}$.

### Neutral squirmers

The reorientation of a neutral swimmer can be immediately understood by examining the instantaneous rotational dynamics. To leading order equation (12) with $B_2=0$ simplifies to

14 $$\begin{aligned} \frac{d \,\theta }{d \,t} = \varepsilon B_1 f(z_c) \sin \theta . \end{aligned}$$

Noting that $B_1$ and $f(z_c)$ are both positive, when $\varepsilon > 0$ we see that $\theta =0$ is an unstable fixed point and all orientations flow towards $\theta =\pm \,\pi$. Conversely, for $\varepsilon < 0$ all orientations flow to $\theta =0$. This means that no matter the orientation or position, the particle is always reorienting to align along ${\textbf{n}} = {\textbf{e}}_z$ and point in the direction of the lower viscosity, consistent with results for squirmers in weak viscosity gradients^40,41^. Because $f\propto z_c^{-4}$, the reorientation rate decreases very quickly with distance from the interface, and thus the reorientation process is ultimately dominated by contact with the interface. One consequence of these dynamics is that a particle going from low to high viscosity can be scattered off the interface depending on its incident orientation.

To quantify the reorientation we note that (13) is separable when $\beta =0$, integrating we obtain

15 $$\begin{aligned} \frac{\sin \theta _f}{\sin \theta _i} = \exp \left[ \frac{3}{2}\varepsilon \int _{z_i}^{z_f}f(z_c)dz_c\right] , \end{aligned}$$

where $\theta _i$ is the orientation at an initial position $z_i$ and likewise $\theta _f$ is the final orientation at $z_f$.

We define the ‘total’ reorientation caused by the interface as the particle crosses from far on one side to far on the other to be the limit when $z_i\rightarrow -\infty$ and $z_f\rightarrow \infty$ (when the particle goes from $\eta _0$ to $\eta _1$). In this case the integral simply equals 1/3 and the total reorientation is given by the formula

16 $$\begin{aligned} \sin \theta _{f} = \exp \left[ \frac{\eta _1-\eta _0}{2\eta _0}\right] \sin \theta _{i}. \end{aligned}$$

This formula bears a striking similarity to Snell’s law of refraction, except here the ‘relative refractive index’ is given by the exponentiated relative viscosity difference. The reorientation is independent of the speed of the particle due to the linearity of the Stokes equations, in this case both the thrust generated by the particle and the drag felt by the particle would be proportional to the $B_1$ mode. This form of reorientation law, $\sin \theta _f=e^\alpha \sin \theta _i$, was found for gliders moving across a substrate featuring a jump in frictional properties^44^. In that case $\alpha = -2a\zeta _{rt}/\zeta _{rr}$ where $\zeta _{rr}$, $\zeta _{rt}$ are torque-rotation and torque-translation resistance coefficients of the particle respectively. The similarities arise because in both cases the particles are subject to linear drag laws.

Unlike the refraction of light, or gliders on a substrate, squirmers interact (hydrodynamically) with the interface from any point in space, but the functional form of the interaction, given by $f(z_c)$ changes upon contact. Because of this we integrate equation (15) in multiple stages, separately accounting for the particle’s approach to the interface $(z_c = -\infty \rightarrow -a$, $\theta = \theta _i \rightarrow \theta _{-a})$, crossing the interface $\left( z_c = -a \rightarrow +a, \theta = \theta _{-a} \rightarrow \theta _a \right)$ and the departure from the interface $(z_c = +a \rightarrow +\infty$, $\theta = \theta _a \rightarrow \theta _f)$ as illustrated in Fig. 3a. In this way we can quantify the starting $\left( \theta _{start} \right)$ and ending $\left( \theta _{end}\right)$ orientation in each of these stages

17 $$\begin{aligned} \sin \theta _{end} = e^{\alpha } \sin \theta _{start}, \end{aligned}$$

where for $\theta _{start} = \left\{ \theta _i, \theta _{-a}, \theta _a \right\}$, and $\theta _{end} = \left\{ \theta _{-a}, \theta _a, \theta _f\right\}$, we find $\alpha = \left\{ \frac{\varepsilon }{32}, \frac{7 \varepsilon }{16}, \frac{\varepsilon }{32}\right\}$, in that order in each of the three stages. We can also relate the particle’s initial and final orientations by combining Eq. (17) in all three stages. We notice that the amount of reorientation during the approach and departure from the interface is the same. However, the large value of $\alpha$ means that the reorientation process is dominated during contact with the interface $\left( \theta _{-a} \rightarrow \theta _a\right)$. And, as discussed earlier, the reorientation process is always in the direction of lower viscosity. In analogy to ray optics, the refraction of the trajectory is always towards the medium of lower resistance. A similar preference was also shown by active particles in linear or diffuse viscosity gradients^24,40,41^, and as we will show below (for pullers), matches well with experimental observations of *Chlamydomonas Reinhardtii* algae swimming across sharp viscosity gradients^25^. In contrast, studies done where the propulsive force is fixed, both for swimmers in diffuse viscosity gradients and gliders across a frictional substrate, show reorientation towards higher viscosities as one might expect due purely to the modulation of drag.

We note that in deriving (16) we assume that the trajectory from one side to the other is physically realizable. This is not always the case. If the active particle is swimming towards a higher viscosity $\eta _1 > \eta _0$, with a sufficiently shallow angle it may be reoriented back (reflected by the interface). But note, due to hydrodynamic interactions, the particle may be reoriented back before even coming into contact with the interface, or even after completely crossing the interface. To examine these phenomena we find the limit of validity of (16), which we define $\theta _i=\theta _{crit}$ which occurs when $\theta _f = \pi /2$, that is the swimmer is tangent to the interface at $z_f\rightarrow \infty$, this case we find

18 $$\begin{aligned} \theta _{crit} = \arcsin \left( \exp \left[ \frac{\eta _0-\eta _1}{2\eta _0}\right] \right) . \end{aligned}$$

Therefore, for $\eta _1>\eta _0$ Eq. (16) is valid only when $\theta _i \le \theta _{crit}$, as a particle with an initial angle, $\theta _i > \theta _{crit}$ (a sufficiently shallow angle of approach to the interface), will be reflected back (it will not reach $z_f\rightarrow \infty$). We can likewise define critical initial angles such that the particle does not cross the interface $\theta _{crit,a} = \arcsin \left( \exp \left[ 15(\eta _0-\eta _1)/(32\eta _0)\right] \right)$, or even touch the interface $\theta _{crit,-a} = \arcsin \left( \exp \left[ (\eta _0-\eta _1)/(32\eta _0)\right] \right)$, using (17). We see that, much like the total reorientation, $\theta _{crit}$ in (18) is dominated by particles which are scattered at the interface, with only a narrow set of initial angles that lead to reflection before or after contact with the interface. Regardless of where the particle is reflected, the entire scattering process is symmetric (about the point when $\theta =\pi /2$ as shown in Fig. 3b) and hence obeys the reflection law

19 $$\begin{aligned} \theta _f = \pi - \theta _i \quad \end{aligned}$$

for all particles when $\theta _i > \theta _{crit}$, as previously shown for gliders^44^.

We have thus far assumed a physically unrealistic discontinuous viscosity change and a simple neutral squirmer in order to derive these simple formulas. In the following sections, we relax these assumptions and find that neither significantly impact the reorientation process.

### Smooth gradients

To investigate the reorientation and scattering of the particle due to a viscosity change that varies smoothly (due to the effects of diffusion), instead of a Heaviside function in (1) we say $H(z)=\left( 1+\tanh (kz)\right) /2$ where $k > 0$ and 1/*k* is the effective length scale over which the viscosity varies between $\eta _0$ and $\eta _1$. Hence, this viscosity variation approaches the discontinuous profile used previously as $k \rightarrow \infty$. Calculation proceeds similarly to that in the sharp gradients. The angular velocity required for this calculation is found by substituting the $\tanh$ viscosity profile in the reciprocal theorem (27) and its expression looks the same as that found in sharp viscosity gradients (10) except for the functions $f\left( z_c \right)$ and $g\left( z_c\right)$ which are given in “Methods” section.

Despite the differences in viscosity profile and angular velocity, we find no difference in the overall reorientation $( \theta _i \rightarrow \theta _f)$ between smooth and sharp viscosity gradients. The law governing the reorientation in smooth gradients is identical to that found in sharp gradients (16). This implies that the critical orientation required for scattering in smooth and sharp viscosity gradients is also the same. It appears that, as with the refraction of light, the interface between fluids of differing viscosity can be smoothed out and the total reorientation remains unchanged. This is surprising, because unlike light, the active particle interacts non-locally with the entire medium at once at all times due to hydrodynamics.

### Pushers and pullers

**Figure 4:**
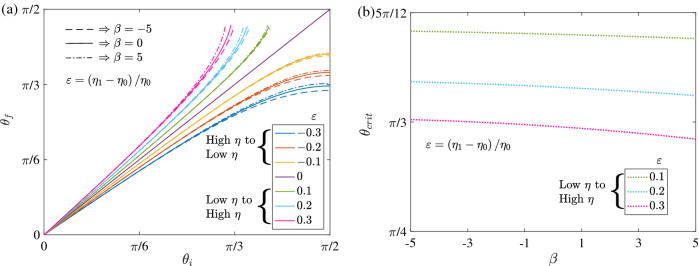
The reorientation of active particles that cross the interface ($\textbf{a}$) and the critical orientation required for scattering from the interface ($\textbf{b}$). Here, the solid, dashed, and dash-dotted lines in ($\textbf{a}$) correspond to the neutral swimmers $\beta = 0$, pushers $\beta = -5$, and pullers $\beta = 5$, respectively. On the other hand, the different line colors in ($\textbf{a}$), ($\textbf{b}$) represent different viscosity jumps $\varepsilon$.

For pushers and pullers the differential equation governing the reorientation (13) is not separable and so we compute the reorientation numerically for $\beta \ne 0$. We find that the reorientation and scattering of pushers or pullers are similar to those of neutral swimmers with only a weak dependence on the squirming ratio $\beta$. See Fig. 4a for the reorientation of the swimmers crossing the interface and Fig. 4b for the critical orientation required for scattering, obtained from the numerical solution of (13). Hence, the pushers and pullers, like the neutral swimmers, orient towards regions of lower viscosity. Going from high to low viscosity ($\varepsilon < 0$) pullers rotate slightly less while the pushers rotate slightly more than the neutral swimmer. Conversely going from low viscosity to high viscosity ($\varepsilon > 0$) pullers rotate more while the pushers rotate less than a neutral swimmer thereby very weakly changing $\theta _{crit}$ as shown in Fig. 4b.

Part of the reason for the weak dependence of reorientation on the $B_2$ mode occurs because the rotation caused by this mode before and after crossing the interface is in the opposite direction (as $g\left( z_c\right)$ is an odd function) and hence counteract each other (they do not cancel due to the cosine term in (13)). Conversely, as discussed previously the rotation caused by the $B_1$ mode is always in the same direction before and after crossing the interface (as $f\left( z_c\right)$ is an even function).

The weak dependence of reorientation on $\beta$ can be leveraged to find the reorientation experienced by the pushers and pullers analytically. This is achieved by expanding the leading order (in $\varepsilon$) orientation $\theta$ in terms of $\beta$ and solving Eq. (13) at each order in $\beta$ as shown in “Methods” section. In principle, such a perturbation holds for only $\left| \varepsilon \right| \ll \left| \beta \right| \ll 1$ but the weak functional dependence yields accurate results up to $\left| \beta \right| \approx 10$ for any $\left| \varepsilon \right| \ll 1$.

### Comparison to experiment

**Figure 5:**
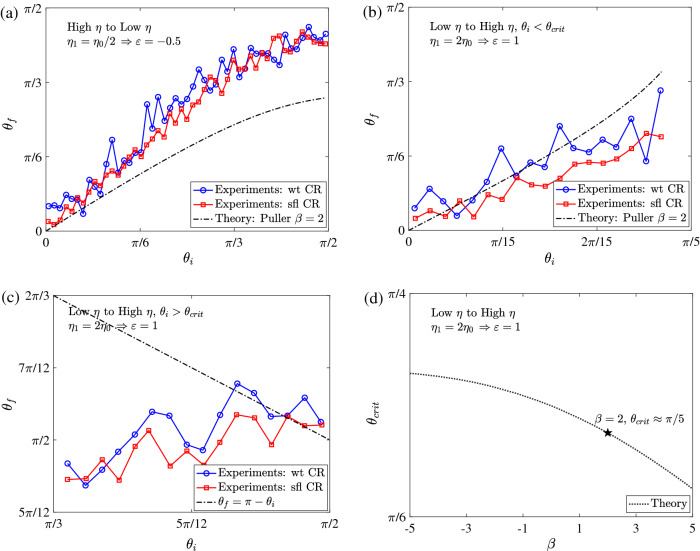
The reorientation of active particles that cross the interface ($\textbf{a}$), ($\textbf{b}$), are reflected by the interface ($\textbf{c}$), and the critical orientation required for scattering from the interface ($\textbf{d}$). Here, the lines with symbols in ($\textbf{a}$), ($\textbf{b}$), and ($\textbf{c}$) represent the previous experiments using wild-type (wt) or short-flagellated (sfl) *Chlamydomonas Reinhardtii*^25^ while the dash-dotted lines correspond to the current theory for pullers $\beta = 2$. Also, the only line in ($\textbf{d}$) represents the current theory.

We now compare our theory with recent experiments conducted with both wild-type (wt) and short-flagellated (sfl) *Chlamydomonas Reinhardtii* (CR), swimming across sharp viscosity gradients^25^. Just as we have predicted above, the CR were found generically to reorient towards lower viscosities and there was a critical angle, going from low to high viscosity, past which the swimmers would be reflected by the viscosity interface. The initial and final orientations of the algae were recorded 1*s* before reaching and 1*s* after crossing the interface in the experiments. Using experimental velocities this equates to $z_i \approx -3a$ and $z_f \approx 3a$ where $a = 5 \upmu$m is the approximate swimmer radius, and we use these values in our theory for comparison. In the experiments, the viscosity of one fluid (water) was held constant ($\eta = 10^{-3}$ Pa s) and a variety of different viscosities were used for the other fluid from 2 to 62$\times$ greater by dissolving varying concentrations of methylcellulose in water, resulting in relative viscosity differences $\left| \varepsilon \right| =$ 0.5–61. We compare our asymptotic theory, which assumes $\varepsilon \ll 1$, only to the smallest values $\varepsilon = -0.5, 1$ representing particle motion from high to low and low to high viscosities respectively. We note that the experimental data indicate small but systematic reorientation even in homogeneous Newtonian fluid ‘control’ experiments. In order to remove this effect we subtracted the reorientation reported in homogeneous fluids from that in finite viscosity gradients and compared the difference with the theory. The experiments reported different swim speeds for wt and sfl algae, but found similar reorientation in viscosity gradients. This is consistent with our theory; our model predicts that the reorientation of a neutral swimmer is independent of speed (see Eqs. (15)–(18)), while the effect of the squirming ratio $\beta$ is very weak for pushers and pullers, as shown in Fig. 4. In light of this, we represent both algae by a single squirmer whose $B_1$ mode is known from the known swim velocity of wt algae in homogeneous fluid $2 B_1/3 \approx 100 \, \upmu$m/s and $B_2$ value from the stresslet exerted by same algae 10 pN$\times 10\,\upmu$m $\approx 4\pi \eta a^2 B_2$ found in other experiments^52^, ultimately yielding the squirming ratio $\beta =2$. Lastly, the algae in experiments displayed diffusive dynamics at large time scales, with translational and rotary diffusivities $D_T$, $D_R$ respectively. But here we neglect both diffusivities as the former does not affect the reorientation while the latter is small compared to angular velocity for the viscosity jumps of interest $\varepsilon = O \left( 1 \right)$, where $D_R \approx 1$ s$^{-1} < \left| {\varvec{\Omega }} \right|$ which is at least 4 s$^{-1}$.

Our theoretical model matches experimental observations reasonably well, in the aforementioned parameter regime. In Fig. 5a, we show the reorientation ($\theta _f$ vs $\theta _i$) for swimming from high to low viscosity ($\varepsilon =-0.5$) while Fig. 5b shows swimming from low to high viscosity ($\varepsilon =1$). Figure 5c shows particles reflected by the viscosity interface. In all cases our model somewhat over-predicts the amount of reorientation but captures nicely the general qualitative features observed in experiments. The largest discrepancy between theory and experiments occurs at shallow angles of approach to the interface $\left( \theta _i > \pi /5 \right)$, when particles undergo large changes in orientation. In particular, experimental data does not well satisfy a symmetric reflection law for particles that are reflected at the interface as shown in Fig. 5c, but the data in this sensitive regime is somewhat limited and further data may clarify this discrepancy. Over-predicting the reorientation naturally leads to a critical angle $\left( \theta _{crit} \approx \pi /5 \right)$ found in theory that is lower than that reported in the experiments $\left( \theta _{crit} = \pi /3 \right)$, where a shallower approach to the interface is needed to scatter (see Fig. 5d).

Quantitative differences between experiments and theory are not surprising as our perturbative approach assumes $\varepsilon \ll 1$ while in experiments at best we have $\varepsilon = O(1)$ . Another possible cause of quantitative discrepancy may be due to confinement of the algae, in a microfluidic channel of height 20 $\upmu$m, in the experiments unlike the free-space assumption made in the theory. We approximated *Chlamydomonas* by a spherical squirmer whose gait remains fixed in viscosity gradients, while in reality alga has a spherical body with two flagella in front, whose beating pattern (or gait) varies with viscosity^25^; we also assumed that the swimmer does not stir the viscosity field due to its motion and any mixing of the fluid in experiments is likely to weaken the effect of viscosity differences on reorientation and we expect these issues in particular to be exacerbated with shallower approachs to interface $\left( \theta _i > \theta _{crit} \right)$, when particles spend a relatively large amount of time close to the interface. Finally, we neglected any density gradients that inevitably exist in viscosity gradients, and which preferentially alter motion of particle going from high to low viscosity (unlike in the opposite direction) due to relatively large volume of fluid dragged by the particle^43^.

## Conclusions

Motivated by the recent experiments showing *Chlamydomonas Reinhardtii* algae scattering at sharp viscosity gradients^25^, we developed a simple analytical model for active particles swimming across sharp changes in the viscosity of the suspending fluid. We found that pushers, pullers and neutral swimmers all interact similarly with the interface. Swimmers are generically reoriented towards the region of lower viscosity (as found in previous studies with weak gradients^40,41^). As a result, if active particles approach a viscosity interface at a sufficiently shallow angle they can be reflected if swimming from low to high viscosity; otherwise, they simply cross the interface undergoing a degree of reorientation set by the relative viscosity difference. This is similar to the refraction or reflection of the light due to a change in refractive index and the law we derive governing the reorientation of neutral swimmers similar to Snell’s law of ray optics (as previously shown for gliders on a frictional substrate^44^). Our theory compares very well with experimental observations^25^ and provides a simple model for the dynamics of active particles in fluids with inhomogeneous viscosity. These results suggest that tailoring the mechanical properties of fluids can be an effective method to control particle speed^53–56^ and orientation^57–60^, ultimately organize active matter systems.

## Methods

### Reciprocal theorem

The dynamics of a force-free and torque-free active particle in a fluid medium of arbitrary rheology is given by^61^

20 $$\begin{aligned} \textbf {\textsf {U}} = {\hat{\textbf{\textsf {R}}}}_{\textbf{\textsf {FU}}}^{-1} \cdot (\textbf{\textsf {F}}_\text{s} + \textbf{\textsf {F}}_{\text{NN}}), \end{aligned}$$

where $\textbf{\textsf {U}}=[{\textbf{U}} \,\varvec{\Omega } ]^\textsf{T}$ is a six-dimensional vector containing the swimmer’s translational and angular velocities, and likewise $\textbf{\textsf {F}} = [{\textbf{F}} \,{\textbf{L}} ]^\textsf{T}$ contains force and torque. $\hat{\textbf{\textsf {R}}}_{\textbf{\textsf {FU}}}$ is the resistance tensor for the particle in a fluid of uniform viscosity $\eta _0$, $\textbf{\textsf {F}}_\text{s}$ is the thrust force and torque due to particle activity in a homogeneous Newtonian fluid of viscosity $\eta _0$, while the additional force $\textbf{\textsf {F}}_{\text{NN}}$ accounts for the changes in the rheological properties (viscosity) of the fluid. The formulas are obtained by using the reciprocal theorem, by projecting onto a known auxiliary flow as an adjoint solution (denoted by a hat),

21 $$\begin{aligned} \textbf{\textsf {F}}_\text{s}& = \int _{S_p} {\textbf{u}}^s \cdot ({\textbf{n}}_p \cdot \hat{\textbf{\textsf {T}}}_{\textbf{\textsf {U}}}) \,dS, \end{aligned}$$

22 $$\begin{aligned} \textbf{\textsf {F}}_{\text{NN}}& = - \int _{\mathscr  {V}} \varvec{\tau }_{NN} : \hat{\varvec{E}}_{\textbf{\textsf {U}}} \,dV, \end{aligned}$$

where $\mathscr  {V}$ denotes the entire fluid volume outside the particle and $\varvec{\tau }_{NN}= \varvec{\sigma } +p{\textbf{I}}-\eta _0\dot{\varvec{\gamma }} = (\eta -\eta _0)\dot{\varvec{\gamma }}$ represents the extra (deviatoric) stress due to changes in viscosity from $\eta _0$. $\hat{\textbf{\textsf {T}}}_{\textbf{\textsf {U}}}$, $\hat{\varvec{E}}_{\textbf{\textsf{U}}}$ are linear operators relating the stress and strain-rate in the fluid to particle velocity, $\hat{\varvec{\sigma }} = \hat{\textbf{\textsf {T}}}_{\textbf{\textsf {U}}} \cdot \hat{\textbf{\textsf {U}}}$ and $\hat{\dot{\varvec{\gamma }}} = 2 \hat{\varvec{E}}_{\textbf{\textsf {U}}} \cdot \hat{\textbf{\textsf {U}}}$ in a fluid of homogeneous viscosity $\eta _0$.

To facilitate the evaluation of swimming velocity in Eq. (20), we assume small relative viscosity differences $\varepsilon \ll 1$ and regular perturbation expansion for any functional dependence on $\varepsilon$, for example $h\left( \varepsilon \right) = h_0 + \varepsilon h_1 + \ldots$. In this way, because the extra stress due to viscosity changes is $O\left( \varepsilon \right)$,

23 $$\begin{aligned} \varvec{\tau }_{NN} = \left( \eta - \eta _0\right) \dot{\varvec{\gamma }} = \varepsilon \eta _0 H\left( z\right) \dot{\varvec{\gamma }} \sim O\left( \varepsilon \right) , \end{aligned}$$

the swimmer is moving through a homogeneous Newtonian fluid of viscosity $\eta _0$ to leading order and its velocity is well known

24 $$\begin{aligned} {\textbf{U}}_0& = \frac{1}{6 \pi \eta _0 a} \int _{S_p} {\textbf{u}}^s \cdot ({\textbf{n}}_p \cdot \hat{\textbf{\textsf {T}}}_{{\textbf{U}}}) \,dS = \frac{2}{3} B_1 {\textbf{p}} , \end{aligned}$$

25 $$\begin{aligned} \varvec{\Omega }_0& = \frac{1}{8 \pi \eta _0 a^3} \int _{S_p} {\textbf{u}}^s \cdot ({\textbf{n}}_p \cdot \hat{\textbf{\textsf {T}}}_{\varvec{\Omega }}) \,dS = \varvec{0}. \end{aligned}$$

The effects of viscosity variations relative to $\eta _0$ are captured at the next order, where the swimming velocity is

26 $$\begin{aligned} {\textbf{U}}_1&= - \frac{1}{6 \pi \eta _0 a} \int _{\mathscr {V}} \varvec{\tau }_{NN,1} : \hat{\varvec{E}}_{{\textbf{U}}}\,dV , \end{aligned}$$

27 $$\begin{aligned} \varvec{\Omega }_1&= - \frac{1}{8 \pi \eta _0 a^3} \int _{\mathscr {V}} \varvec{\tau }_{NN,1} :\hat{\varvec{E}}_{\varvec{\Omega }}\,dV . \end{aligned}$$

Here, $\varvec{\tau }_{NN,1} = \eta _0 H\left( z\right) \dot{\varvec{\gamma }}_0$ and $\dot{\varvec{\gamma }}_0 = \nabla {\textbf{u}}_0 + (\nabla {\textbf{u}}_0)^T$ is the rate of strain tensor associated with the leading order flow ${\textbf{u}}_0$. These small viscosity variations $\varepsilon \eta _0 H\left( z\right)$ alter the velocity of swimmer in homogeneous fluid ${\textbf{U}}_0$, $\varvec{\Omega }_0$ by a small correction ${\textbf{U}}_1$, $\varvec{\Omega }_1$. An evaluation of integrals in Eqs. (26), (27) with discontinuous viscosity jump (*H* is a Heaviside function) yields this correction as

28 $$\begin{aligned} {\textbf{U}}_1&= B_1 \left( A(z_c) {\textbf{n}} + B(z_c) {\textbf{p}} \right) + B_2 \left( C(z_c) {\textbf{n}} + D (z_c) (\textbf{pp} \cdot {\textbf{n}}) + E (z_c) ({\textbf{n}} \cdot {\textbf{p}})^2 {\textbf{n}} \right) , \end{aligned}$$

29 $$\begin{aligned} \varvec{\Omega }_1&= \bigl (B_1 f(z_c) + B_2 g(z_c) ({\textbf{n}} \cdot {\textbf{p}}) \bigr ) ({\textbf{n}} \times {\textbf{p}}), \end{aligned}$$

where for $$\left| z_c\right| >a$$

30 $$\begin{aligned} A\left( z_c\right)= & {} \frac{a^3(-a^2 + 3 z_c^2)}{24 z_c^{5}},\,B\left( z_c\right) = \frac{a^3(-a^2 + z_c^2)}{24 z_c^{5}},\,C\left( z_c\right) = \frac{a^2(-5a^4 + 12 a^2 z_c^2 - 9 z_c^4) }{96 z_c^{6}},\nonumber \\ D\left( z_c\right)= & {} \frac{a^2(5a^4 -9 a^2 z_c^2 + 9 z_c^4) }{48 z_c^{6}},\,E\left( z_c\right) = \frac{a^2(5a^4 - 18 12 a^2 z_c^2 + 9 z_c^4) }{96 z_c^{6}}, \end{aligned}$$

31 $$\begin{aligned} f\left( z_c\right)= & {} \frac{a^3}{16 z_c^4},\,g\left( z_c\right) = \frac{(-4a^2 + 3z_c^2)a^2}{32 z_c^5}, \end{aligned}$$

and for $$\left| z_c\right| \le a$$

32 $$\begin{aligned} A\left( z_c\right)= & {} \frac{ z_c(3a^2 - z_c^2)}{24 a^3},\,B\left( z_c\right) = \frac{ z_c(a^2 - z_c^2)}{24 a^3},\,C\left( z_c\right) = \frac{-5a^4 + 6 a^2 z_c^2 - 3 z_c^4}{96a^4},\nonumber \\ D\left( z_c\right)= & {} \frac{8 a^4 -3 z_c^4}{48 a^4},\,E\left( z_c\right) = \frac{-a^4 - 18 a^2 z_c^2 + 15 z_c^4}{96a^4}, \end{aligned}$$

33 $$\begin{aligned} f\left( z_c\right)= & {} \frac{3a^2 - 2 z_c^2}{16 a^3},\,g\left( z_c\right) = \frac{(-7a^2 + 6 z_c^2)z_c}{32 a^4}. \end{aligned}$$

When we assume a smooth viscosity profile $H(z)=\left( 1+\tanh (kz)\right) /2$, we obtain

34 $$\begin{aligned} f (z_c)&= \int _{-\infty }^{-a} \frac{a^3 \{ 1+ \tanh [k(z+z_c)] \}}{8z^5} d z + \int _{-a}^{a} \frac{z \{ 1+ \tanh [k(z+z_c)] \}}{8a^3} d z + \int _{a}^{\infty } \frac{a^3 \{ 1+ \tanh [k(z+z_c)] \}}{8z^5} d z, \end{aligned}$$

35 $$\begin{aligned} g (z_c)&= \int _{-\infty }^{-a} \frac{(20a^4 - 9a^2z^2) \{ 1+ \tanh [k(z+z_c)] \}}{64z^6} d z + \int _{-a}^{a} \frac{(18z^2 -7 a^2) \{ 1+ \tanh [k(z+z_c)] \}}{64a^4} d z \nonumber \\&\quad + \int _{-\infty }^{-a} \frac{(20a^4 - 9a^2z^2) \{ 1+ \tanh [k(z+z_c)] \}}{64z^6} d z. \end{aligned}$$

### Pushers and pullers, the effect of $\beta$

In order to find the leading order effect of the second squirming mode for pushers and pullers, we assume $\beta \ll 1$ and perform a regular perturbation expansion of the orientation in $\beta$

36 $$\begin{aligned} \theta = \theta _0 + \theta _1 \beta + O(\beta ^2, \varepsilon ). \end{aligned}$$

Here, we assumed $\left| \beta \right| \gg \left| \varepsilon \right|$ and retained the terms at $O\left( \beta \right)$ unlike those at $O\left( \varepsilon \right)$. We substitute this expansion in (13) and solve the resulting equation at each order in $\beta$. At zeroth order, pushers or pullers become neutral swimmers and the orientation is given by (16). Any deviations relative to the reorientation of the neutral swimmer are captured at the next order where

37 $$\begin{aligned} \frac{d \,\theta _1}{d \,z_c} - \frac{3\varepsilon }{2} \frac{f(z_c)}{\cos ^2 \theta _0} \theta _1 = \frac{3}{2} \varepsilon g(z_c) \sin \theta _0. \end{aligned}$$

The initial condition is $\theta _1 = 0$ as $z_c \rightarrow -\infty$. We solve (37) in multiple stages, separately accounting for the reorientation during the interface approach, crossing and departure. We find

38 $$\begin{aligned} \left. \theta _1 \right| _{z_c = -a}&= \frac{G_1}{\sqrt{e^{-\frac{\varepsilon }{16}} - \sin ^2 \theta _i}},\nonumber \\ G_1&= \int _{-\infty }^{-1} \varepsilon \sin \theta _i \sqrt{1 - e^{\frac{\varepsilon }{16 z^3}} \sin ^2 \theta _i} \frac{\left( - 12 + 9 z^2\right) }{64 z^5} d z. \end{aligned}$$

as the particle touches the interface. Then

39 $$\begin{aligned} \left. \theta _1 \right| _{z_c = a}&= \frac{ G_2 + G_1 }{\sqrt{e^{\frac{-15\varepsilon }{16}} - \sin ^2 \theta _i}},\nonumber \\ G_2&= \int _{- 1}^{1} \varepsilon \sin \theta _i \sqrt{1 - e^{\frac{\varepsilon (8 + 9 z - 2 z^3)}{32}}\sin ^2 \theta _i} \frac{\left( -21 z + 18 z^3\right) }{64} d z, \end{aligned}$$

as the particle crosses the interface and eventually to

40 $$\begin{aligned} \theta _{1f}&= \left. \theta _1 \right| _{z_c \rightarrow \infty } = \frac{ G_3 + C }{\sqrt{1 - e^{\varepsilon }\sin ^2 \theta _i}}, \nonumber \\ G_3&= \int _{1}^{\infty } \varepsilon \sin \theta _i e^{ \frac{\varepsilon }{2}}\sqrt{1 - e^{\varepsilon (1 -\frac{1}{16 z^3})}\sin ^2 \theta _i} \frac{\left( - 12 + 9 z^2\right) }{64 z^5} d z, \nonumber \\ C&= \sqrt{\frac{e^{\frac{\varepsilon }{16}} - e^{\varepsilon }\sin ^2 \theta _i}{e^{-\frac{15}{16}\varepsilon } - \sin ^2 \theta _i}} (G_2 + G_1), \end{aligned}$$

as the particle departs away from the interface. Accounting for the leading order reorientation, the final orientation of pushers or pullers as they cross and go far ahead of the interface is

41 $$\begin{aligned} \theta _f = \theta _{0f} + \beta \theta _{1f} + O\left( \beta ^2, \varepsilon \right) , \end{aligned}$$

where $\theta _{0f} = \left. \theta _0 \right| _{z_c \rightarrow \infty }$ follows from (16) as $\sin \theta _{0f} = e^{\varepsilon /2} \sin \theta _i$.

## Data Availability

The datasets used and/or analysed during the current study are available from the corresponding author on reasonable request.
