# Publisher Correction: Active particles crossing sharp viscosity gradients

**DOI:** 10.1038/s41598-023-30272-0

**Published:** 2023-02-22

**Authors:** Jiahao Gong, Vaseem A. Shaik, Gwynn J. Elfring

**Affiliations:** 1 Department of Mathematics, University of British Columbia, 1984 Mathematics Road, Vancouver, BC V6T 1Z2 Canada; 2 Department of Mechanical Engineering, Institute of Applied Mathematics, University of British Columbia, Vancouver, BC V6T 1Z4 Canada

Correction to: *Scientific Reports* https://doi.org/10.1038/s41598-023-27423-8, published online 11 January 2023

The original HTML version of this Article contained formatting errors in the Methods, under the subheading ‘Reciprocal theorem’, where the Equations 20, 21, 22, 24 and 25 and the formulas in the respective text underneath Eq. 20 and Eq. 22 were incorrectly formatted.

The original Article has been corrected.

